# Conjunctival Inflammatory Gene Expression Profiling in Dry Eye Disease: Correlations With HLA-DRA and HLA-DRB1

**DOI:** 10.3389/fimmu.2018.02271

**Published:** 2018-10-15

**Authors:** Karima Kessal, Hong Liang, Ghislaine Rabut, Philippe Daull, Jean-Sébastien Garrigue, Mylene Docquier, Stéphane Melik Parsadaniantz, Christophe Baudouin, Françoise Brignole-Baudouin

**Affiliations:** ^1^Sorbonne Université, UPMC Univ Paris 06, INSERM, CNRS, Institut de la Vision, Paris, France; ^2^Department of Ophthalmology III, Quinze-Vingts National Ophthalmology Hospital, Paris, France; ^3^Quinze-Vingts National Ophthalmology Hospital, DHU Sight Restore, INSERM-DGOS CIC 1423, Paris, France; ^4^Santen SAS, Evry, France; ^5^iGE3 Genomics Platform University of Geneva, Geneva, Switzerland; ^6^Department of Ophthalmology, Ambroise Paré Hospital, APHP, University of Versailles Saint-Quentin en Yvelines, Boulogne-Billancourt, France; ^7^Sorbonne Paris Cité Université Paris Descartes, Faculté de Pharmacie de Paris, Paris, France

**Keywords:** HLA-DR, inflammatory targets, NanoString^®^ assay, conjunctival imprints, dry eye disease

## Abstract

**Purpose:** In several multicenter clinical trials, HLA-DR was found to be a potential biomarker of dry eye disease (DED)'s severity and prognosis. Given the fact that HLA-DR receptor is a heterodimer consisting in an alpha and a beta chain, we intended to investigate the correlation of inflammatory targets with the corresponding transcripts, *HLA-DRA* and *HLA-DRB1*, to characterize specific targets closely related to HLA-DR expressed in conjunctival cells from patients suffering from DED of various etiologies.

**Methods:** A prospective study was conducted in 88 patients with different forms of DED. Ocular symptom scores, ocular-staining grades, tear breakup time (TBUT) and Schirmer test were evaluated. Superficial conjunctival cells were collected by impression cytology and total RNAs were extracted for analyses using the new NanoString^®^ nCounter technology based on an inflammatory human code set containing 249 inflammatory genes.

**Results:** Two hundred transcripts were reliably detected in conjunctival specimens at various levels ranging from 1 to 222,546 RNA copies. Overall, from the 88 samples, 21 target genes showed a highly significant correlation (*R* > 0.8) with *HLA-DRA* and *HLA-DRB1, HLA-DRA* and *B1* presenting the highest correlation (*R* = 0.9). These selected targets belonged to eight family groups, namely interferon and interferon-stimulated genes, tumor necrosis factor superfamily and related factors, Toll-like receptors and related factors, complement system factors, chemokines/cytokines, the RIPK enzyme family, and transduction signals such as the STAT and MAPK families.

**Conclusions:** We have identified a profile of 21 transcripts correlated with *HLA-DR* expression, suggesting closely regulated signaling pathways and possible direct or indirect interactions between them. The NanoString^®^ nCounter technology in conjunctival imprints could constitute a reliable tool in the future for wider screening of inflammatory biomarkers in DED, usable in very small samples. Broader combinations of biomarkers associated with HLA-DR could be analyzed to develop new diagnostic approaches, identify tighter pathophysiological gene signatures and personalize DED therapies more efficiently.

## Introduction

The definition of dry eye disease (DED) has recently been revised to “a multifactorial disease of the ocular surface characterized by a loss of homeostasis of the tear film, and accompanied by ocular symptoms, in which tear film instability and hyperosmolarity, ocular surface inflammation and damage, and neurosensory abnormalities play etiological roles” (1).

Indeed, among the DED definition criteria, its pathogenesis has been largely described as the result of chronic inflammation and activation of the immune system, with involvement of a wide variety of inflammatory mediators, notably chemokines and cytokines (2–5). These markers have been explored in either tears or the conjunctiva (6), such as: Human leukocyte antigen-DR (HLA-DR) (7, 8), Interleukins (ILs): IL-6, IL-1α, IL-8 (9), IL-1β, Matrix Metalloproteinases-9 (MMP-9) (10), Interferon-gamma (IFN-γ), IL-17, and CXCL10 (11).

HLA-DR is a transmembrane heterodimer consisting of alpha (α) and beta (β) glycoprotein chains, and belonging to the major histocompatibility complex (MHC) class II receptors. The α and β chains are encoded by separate genes and their expressions are exquisitely controlled at the transcriptional level (12).They are constitutively expressed by antigen-presenting cells (APCs), such as macrophages, B-lymphocytes, and dendritic cells, but they can also be induced in activated T-lymphocytes and non-professional APCs such as epithelial cells in inflammatory conditions (13).

Using flow cytometry, a technique designed to quantify both levels of expression of a marker by a cell population and the number of cells bearing the targeted protein, HLA-DR was detected in conjunctival epithelial cells obtained by conjunctival imprints (CIs) from DED patients (7). It was reported to be associated with disease severity (14) and correlated with symptoms and signs as corneal fluorescein staining (13). Several multicenter trials have included this methodology as a tool evaluating ocular surface (OS) inflammation (13, 15). Therefore, HLA-DR is now considered as one of the most promising markers of OS inflammation (16, 17).

To better understand the regulatory loop of HLA-DR expression and to investigate the specific inflammatory targets associated with HLA-DR induction, a transcriptomic and multiplexed approach was used on the CIs of ocular surface disease (OSD) patients. Additionally, as the (α/β) heterodimers are the main products of the HLA-DRA and B1genes, respectively, investigations of the gene transcripts correlated with them can contribute to a better understanding of the transcriptional activation and regulation of HLA-DR's complex expression.

Previous transcript analyses were mainly carried out using detection methods as classical PCR, qPCR, RNA-Seq, or microarray (9, 18, 19). In contrast to RNA-Seq, which is based on sequenced RNA converted to a cDNA library, the microarray method is based on the direct detection of the hybridized RNA with labeled probes. Although microarray and RNA-Seq are two major high-throughput technologies for studying RNA expression, these techniques suffer from certain disadvantages such as the presence of background noise. NanoString^®^ technology combines the advantages of both microarray and RNA-Seq with a high resolution and a low level of background noise. It uses digital color-coded bar probes to ensure a multiplexed measurement of gene expression (20). This quantitation method offers a high level of accuracy and sensitivity of individual transcript counts without enzymatic reactions, specifically with a minimal amount of total RNA (21). It is a powerful gene screening technology, used for determining gene expression profiles and has application in molecular-level diagnosis analysis, in several diseases (22).

In this study, we therefore used this new NanoString^®^ nCounter technology to characterize specific inflammatory targets associated with HLA-DR in order to identify related signaling pathways triggered in a conjunctival inflammatory context in DED patients regardless of their underlying etiology.

## Materials and methods

### Clinical evaluation and specimen collection

This prospective single-center study was conducted from January 2014 to December 2015 at the Clinical Investigation Centre (CIC INSERM 1423) of the Quinze-Vingts National Ophthalmology Hospital. The study was conducted in accordance with the Declaration of Helsinki (1964) and approved by the CPP–Ile-de-France V Ethics Committee (number 10793). All patients were informed of the aim and methods of the study and gave their consent. The aim of the study was to examine the gene correlation levels with HLA-DR in a DED patient population without consideration on their etiology. In this study, 88 patients were included, 19 males and 69 females, suffering from various causes of DED: primary Sjögren syndrome (pSS, *n* = 30), meibomian gland dysfunction (MGD, *n* = 41), allergy-related DED (*n* = 7), iatrogenic disorders (*n* = 5), and graves' disease (*n* = 5). Conjunctival superficial cells were collected using application of a polyether sulfone filter (Supor^®^, Gelman, Pall Science, Ann Arbor, MI, USA) onto the anesthetized bulbar conjunctiva and immediately put into a 2-mL plastic dry tube and stored at (−80°C) until use.

### RNA isolation from conjunctival imprint cells and quality measurement

Total RNAs were extracted from conjunctival cells using an RNA-XS kit from Macherey-Nagel. RNA yield and purity were assessed using NanoDrop ND-100 Spectrophotometer (NanoDrop technologies, Rockland, DE, USA). The RNA purity was assessed using the absorbance ratio between RNA and proteins, read at 260 and 280 nm, respectively (A260/280). Total RNA integrity was evaluated with the Agilent 2100 bioanalyzer (Agilent Technologies, Wilmington, DE, USA) according to the manufacturer's specifications.

An RNA integrity number (RIN) greater than 8 was considered as an acceptable quality criterion for the analysis. The instrument software generates a RIN score based on its entire electropherogram. RIN values range from 1 to 10, from a totally degraded RNA to the highest-quality RNA. A cut-off of RIN = 8.0 was used to ensure good RNA quality. RNA from CIs shows a high quality with a RIN greater than 8 for all samples. Total RNA, with a high RNA quality and purity (A260/280 = 1.8; RIN > 8), isolated from conjunctival cells collected from the 88 DED patients was used for quantitative analysis using the inflammatory NanoString^®^ CodeSet panel.

### Nanostring® ncounter assay

The gene expression panel (Table 1) was measured in conjunctival cells using a multiplexed hybridization assay and specific fluorescent barcode probes with no amplification step. Inflammatory gene expression was measured with nCounter^®^ human Inflammation v2 CodeSet^1^ Technologies, Seattle, WA, USA) on the NanoString^®^ nCounter analysis system (NanoString Technologies).

**Table 1:** Gene expression CodeSet panel analyzed using nCounter^®^ Human Inflammation v2.

**Functional family**	***n***	***Gene name***
MHC and cell surface receptor	11	*HLADRA, HLADRB1, CD4, CD86, CD163, AGER, TREM2, FXYD2, MRC1, TBXA2R, TYROBP*
IFN and ISGs	16	*IFNA1, IFNB1, IFNG, IFI44, IFIT1, IFIT2, IFIT3, IRF1, IRF3, IRF5, IRF7, HSH2D, MX1, MX2, OAS2, OASL*
TNF superfamily	11	*TNF, LTA, LTB, CD40LG, FASLG, TNFSF14, CD40, TRAF2, TNFAIP3, TRADD, BIRC2*
Chemokines and receptors	31	*CCL2, CCL3, CCL4, CCL5, CCL7, CCL8, CCL11, CCL13, CCL16, CCL17, CCL19, CCL20, CCL21, CCL22, CCL23, CCL24, CXCL1, CXCL2, CXCL3, CXCL5, CXCL6, CXCL9, CXCL10, CCR1, CCR2, CCR3, CCR4, CCR7, CXCR1, CXCR2, CXCR4*
Interleukins and receptors	30	*IL1A, IL1B, IL2, IL3, IL4, IL5, IL6, IL7, IL8, IL9, IL10, IL11, IL12A, IL12B, IL13, IL15, IL17A, IL18, IL21, IL22, IL1RN, IL6R, IL10RB, IL22RA2, IL23A, IL23R, IL1R1, IL1RAP, IL18RAP, TSLP*
Prostaglandin receptors	9	*PTGDR2, PTGER1, PTGER2, PTGER3, PTGER4, PTGFR, PTGIR, PTGS1, PTGS2*
Toll-like receptors	9	*TLR1, TLR2, TLR3, TLR4, TLR5, TLR6, TLR7, TLR8, TLR9*
Growth factor	1	*AREG*
TGF	4	*TGFB1, TGFB2, TGFB3, TGFBR1*
VEGF	1	*FLT1*
Leukotriene receptors	4	*LTB4R, LTB4R2, CYSLTR1, CYSLTR2*
Complement and CRP	20	*C1QA, C1QB, C1R, C1S, C2, C3, C4A, C5, C6, C7, C8A, C8B, C9, CFB, CFD, CD55, C3AR1, ITGB2, MASP1, MASP2*
GTPase family	5	*RAPGEF2, HRAS, RHOA, CDC42, RAC1*
Serine/threonine kinase	6	*LIMK, PRKCA, PRKCB, RIPK1, RIPK2, ROCK2*
Tyrosine kinase	2	*PTK2, RPS6KA5*
Enzymes	12	*ALOX12, ALOX15, ALOX5, ARG1, BCL2L1, HDAC4, NOS2, NOX1, OASL, PLA2G4A, PLCB1, PPP1R12B*
G-Protein subunit	5	*GNB1, GNGT1, GNAQ, GNAS, OXER1*
HSP family	2	*HSPB1, HSPB2*
NOD-family	2	*NOD1, NOD2*
Adaptor proteins	9	*GRB2, KEAP1, LY96, MBL2, MYD88, MYL2, PDGFA, SHC1, TOLLIP*
Co-factors	6	*CFL1, CRP, DAXX, DEFA1, KNG1, NLRP3*
CSF	3	*CSF1, CSF2, CSF3*
MMP	2	*MMP3, MMP9*
Transcription factors	3	*ATF2, BCL6, CEBPB, CREB1, DDIT3, ELK1, FOS, HIF1A, HMGB1, HMGB2, HMGN1, JUN, MAFF, MAFG, MAFK, MAX, MEF2A, MEF2BNB, MEF2C, MEF2D, MYC, NFATC3, NFE2L2, NFKB1, NR3C1, RELA, RELB, SMAD7, TCF4, TWIST2*
MAPK	15	*MAP2K1, MAP2K4, MAP2K6, MAP3K1, MAP3K5, MAP3K7, MAP3K9, MAPK1, MAPK14, MAPK3, MAPK8, MAPKAPK2, MAPKAPK5, MKNK1, RAF1*
STAT family	3	*STAT1, STAT2, STAT3*
Endogenous genes	6	*CLTC, GAPDH, GUSB, HPRT1, PGK1, TUBB*

*The 249 genes were classified according to their family and interacting factors. The CodeSet includes six internal reference genes. MHC II, Major histocompatibility complex class II; IFN, interferon; ISGs, IFN-stimulating genes; TNF, tumor necrosis factor; CRP, complement regulatory proteins; GTPase, guanosine triphosphatase; CFS, colony-stimulating factors; MMP, matrix metalloproteinases; MAPK, mitogen-activated protein kinases; STAT, signal transducer activator transcription; HSP, heat shock protein; NOD, nucleotide binding oligomerization domain*.

The code set is constituted of biotinylated capture probes and reporter probes attached to color barcode tags for the 249 inflammation-related human genes and six internal reference genes (Table 1). Briefly, purified RNA was diluted in nuclease-free water to 20 ng/μL, making a final assay concentration of 100 ng. Samples were incubated 16–22 h at 65°C as per the manufacturer's standard protocol to ensure hybridization with reporter and capture probes. After hybridization, the samples were processed in the Prep Station and counted in the digital analyzer.

### Nanostring data analysis

The number of counts from RCC files of each gene in the CodeSet was analyzed using Microsoft Excel software. The number of transcript copies was then normalized using the geometric mean of six reference genes and was log2-transformed for further analysis.

### Statistical analysis

The correlations between the different inflammatory mRNA counts were evaluated with the Spearman correlation test (*R*) using Graph Pad Prism 7.0 software; *R* > 0.8 was considered as an appropriate correlation level allowing the selection of targets of interest for accurate gene profiling.

## Results

### Inflammatory gene expression in conjunctival cells from DED patients

NanoString^®^ nCounter analysis covering 249 target genes was used among the 38-gene families (Table 1). Two hundred out of the 249 genes analyzed were detected with more than 50 copies, whereas 49 genes (Table 2) were not detected or were below 50 RNA copies per sample. However, the genes corresponding to the family of Tumor Necrosis Factor (TNF) receptors, Mitogen Activated Protein Kinase (MAPK), and Signal Transducer and Activator of Transcription (STAT) families were detected in all DED patient sub groups.

**Table 2:** List of undetected inflammatory genes in conjunctival cells from CI samples collected in DED patients.

	***n***	**Gene name**
MHC and cell surface receptor	2	*FXYD2, TBXA2R*
IFN and ISGs	2	*IFNA1, IFNB1*
Chemokines and receptors	9	*CCL2, CCL7, CCL8, CCL11, CCL16, CCL19, CCL21, CCL23, CCR3*
Interleukins and receptors	9	*IL1A, IL2, IL9, IL10, IL11, IL12B, IL13, IL21, TSLP*
Prostaglandin receptors	4	*PTGIR, PTGS1, PTGER3, PTGDR2*
Toll-like receptors	2	*TLR7, TLR9*
TGF	2	*TGFB2, TGFB3*
VEGF	1	*FLT1*
Leukotrien receptors	1	*CYSLTR2*
Complement and CRP	8	*C5, C6, C7, C8A, C8B, C9, MASP1, MASP2*
Enzymes	2	*ALOX12, ARG1*
Adaptor proteins	2	*MBL2, MYL2*
Co-factors	1	*KNG1*
CSF	1	*CSF2*
MMP	1	*MMP3*
Transcription factors	2	*MEF2B,TWIST2*

*A total RNA of 100 ng was analyzed on the nCounter^®^ human inflammation v2 chip containing 249 unique fluorescent barcoded probes to detect mRNA abundance. Forty-nine genes were not detected. Genes were classified according to their family*.

### Correlations of detected inflammatory mediators with both *HLA-DRA/B1* receptor transcripts

**Figure 1:**
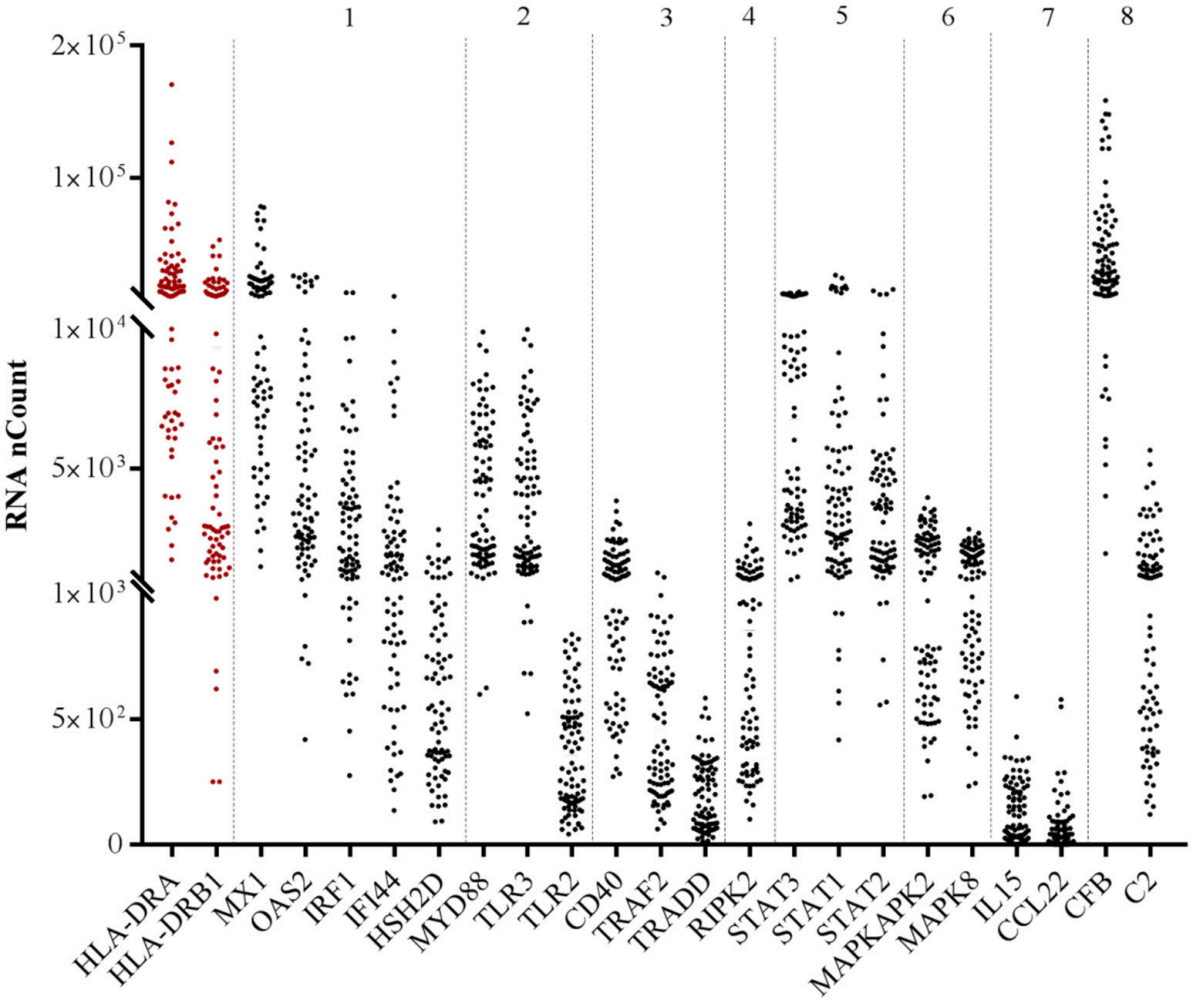
RNA abundance of the selected highly correlated genes with both *HLA-DR* transcripts according to their family. RNA abundance is represented by the detected RNA copies on the y-axis. Numbers above the hatched rectangle correspond to each target family selected: (1) IFN and ISGs, (2) TNF superfamily, (3) TLR and related factors, (4) chemokines/cytokines, (5) complement and CRP (6) RIPK enzymes, (7) STAT, (8) MAPK.

We next investigated the pairwise Spearman correlation among the 200 genes detected and their relationships with both HLA-DR receptors *HLA-DRA* and *HLA-DRB1*. Of the 200 genes detected, 21 displayed correlations higher than 0.8 with both *HLA-DR* (Tables 3A,B). The related inflammatory transcripts included: *IRF1, IFI44, HDH2D, Mx1, OAS2, CD40, TRAF2, TRADD*,*TLR2, TLR3, MyD88, CL22, IL15, C2, CFB, RIPK2, STAT1, STAT2, STAT3, MAPK8*, and *MAPKAP2*, with a highly positive and significant correlation (*R* > 0.8, *p* < 0.0001^***^). These inflammatory targets belonged to eight major families: (1) IFN and interferon-stimulating genes (ISGs), (2) TNF superfamily, (3) the receptor interacting protein kinase family (RIPK), (4) chemokines/cytokines, (5) toll-like receptors, (6) complement and complement regulatory proteins (CRPs), (7) STAT (8) MAPK families. Finally, *HLA-DRA* and *HLA-DRB1* displayed a very high significant correlation between them (*R* = 0.90, *p* < 0.0001^***^). Figure 1 shows the distribution of each gene on the whole sample population as related to its family.

**Table 3:** Selected inflammatory targets displaying a high correlation with *HLA-DRA* and *HLA-B1* in conjunctival cells.

**Genes**	**Gene name**	***HLA-DRA***		***HLA-DRB1***	
		***R***	***p***	***R***	***p***
**(A) OF THE 200 DETECTED GENES, 21 TARGETS DISPLAY A HIGH CORRELATION (*****R*** > **0.8) WITH BOTH *****HLA-DR***** TRANSCRIPTS**					
*HLA-DRB1*	Major histocompatibility complex, class II, DR alpha	0.90	***		
*HLA-DRA*	Major histocompatibility complex, class II, DR beta 1			0.90	***
**IFN AND ISGs**					
*IRF1*	IFN regulatory factor 1	0.88	***	0.89	***
*IFI44*	Interferon-induced protein 44	0.84	***	0.84	***
*HSH2D*	Hematopoietic SH2 domain containing	0.83	***	0.87	***
*MX1*	Myxovirus (influenza virus) resistance 1	0.85	***	0.86	***
*OAS2*	2′-5′-oligoadenylate synthetase 2	0.82	***	0.82	***
**Toll-Like Receptors and Related Factors**					
*TLR2*	Toll-like receptor 2	0.82	***	0.82	***
*TLR3*	Toll-like receptor 3	0.82	***	0.78	***
*MYD88*	Myeloid differentiation primary response gene (88)	0.80	***	0.84	***
**TNF Superfamily**					
*CD40*	CD40 molecule	0.84	***	0.84	***
*TRAF2*	TNF receptor-associated factor 2	0.80	***	0.86	***
*TRADD*	TNFRSF1A-associated via death domain	0.81	***	0.85	***
**Enzymes**					
*RIPK2*	Receptor-interacting serine-threonine kinase 2	0.84	***	0.85	***
**Chemokines/cytokines**
*CCL22*	Chemokine (C-C motif) ligand 22	0.80	***	0.76	***
*IL15*	Interleukin 15	0.80	***	0.84	***
**Complement and CRP**					
*C2*	Complement component 2	0.88	***	0.89	***
*CFB*	Complement factor B	0.83	***	0.82	***
**STAT**					
*STAT1*	Signal transducer and activator of transcription 1	0.89	***	0.88	***
*STAT2*	Signal transducer and activator of transcription 2	0.86	***	0.90	***
*STAT3*	Signal transducer and activator of transcription 3	0.85	***	0.87	***
**MAPK**					
*MAPK8*	Mitogen-activated protein kinase 8	0.82	***	0.82	***
*MAPKAPK2*	Mitogen-activated protein kinase-activated protein kinase 2	0.81	***	0.82	***

Spearman's rank-order correlation test was carried out; p < 0.001***.

**Table:** 

**Genes**	**Family/functions**	**References**
**(B) CHARACTERISTICS OF SELECTED GENES ACCORDING TO THEIR FAMILY AND FUNCTIONS**		
*HLA-DRA*	Alpha chain of the heterodimer MHC class II (α, β)/cell-surface glycoproteins/APCs	(8, 23)
*HLA-DRB1*	Beta chain of the heterodimer MHC class II (α, β)/cell-surface glycoproteins/APCs	(8, 18, 23)
**IFN AND ISGs**		
*IRF1*	Type I, II IFN/interferon regulatory transcription factor family/regulating apoptosis	(24)
*IFI44*	Type I, IFN/viral response/antiproliferative/associated with hepatitis C virus infection	(25–27)
*HSH2D*	Type I, IFN/intracellular protein tyrosine kinase signaling, regulation of cytokine signaling and cytoskeletal reorganization	(28, 29)
*MX1*	Type I, III IFN/GTPases/viral response/programmed cell death regulation of apoptosis	(30–32)
*OAS2*	Type I, III IFN/2–5 A synthetase family/viral response/degradation of viral RNA	(33, 34)
**Toll-like Receptor and Related**		
*TLR2*	Cell-surface protein/pathogen recognition/innate immunity/apoptosis	(35–38)
*TLR3*	Cell-surface protein/recognizes viral dsRNA/innate immunity/apoptosis, NF-κB activation/production of type I IFN	(35, 38, 39)
*MYD88*	Cytosolic adapter protein/innate and adaptive immune response/interleukin-1 and Toll-like receptor signaling pathways.	(38)
**TNF Superfamily**		
*CD40*	TNF receptor superfamily member 5/adaptive immune response/TNFR/membrane receptor/regulation of immune reactions	(40, 41)
*TRAF2*	Adaptor molecule/p38, Akt and JNK activation	(42, 43)
*TRADD*	Adaptor molecule/apoptosis/cell death signaling and NF-κB activation	(44)
**Enzymes**		
*RIPK2*	NF-κB activation/apoptosis/innate and adaptive immune pathways	(45, 46)
**MAPK**		
*MAPKAPK2*	Kinases/MAPKs subtype p38/regulation of pro-inflammatory cytokines	(47, 48)
*MAPK8*	Kinases/stress-responsive c-Jun N-terminal kinase (JNK)/proliferation, differentiation, transcription regulation	(49)
**STAT**		
*STAT1*	Activators of transcription/cell viability/response to IFN	(50)
*STAT2*	Activators of transcription/cell viability/response to IFN	(50)
*STAT3*	Activators of transcription/cell viability/response to IFN	(50)
**Chemokines/Cytokines**		
*CCL22*	Ligand of chemokine receptor CCR4/Th2 cell migration	(51)
*IL15*	Type-I cytokine family/regulates T and natural killer cell activation and proliferation/activation of JAK kinases/not secreted/monocytes/macrophages	(52)
**Complement AND CRP**		
*C2*	Extracellular region/classical pathway of complement activation/innate immunity	(53)
*CFB*	Extracellular region/alternative pathway of complement activation/innate immunity	(54)

*The selected genes showing high correlations with R > 0.8 are described according to their family and their function in various cells*.

### Differential distribution of inflammatory genes in patients with sjögren syndrome dry eye (SSDE) and non-sjögren syndrome dry eye (NSSDE)

**Figure 2:**
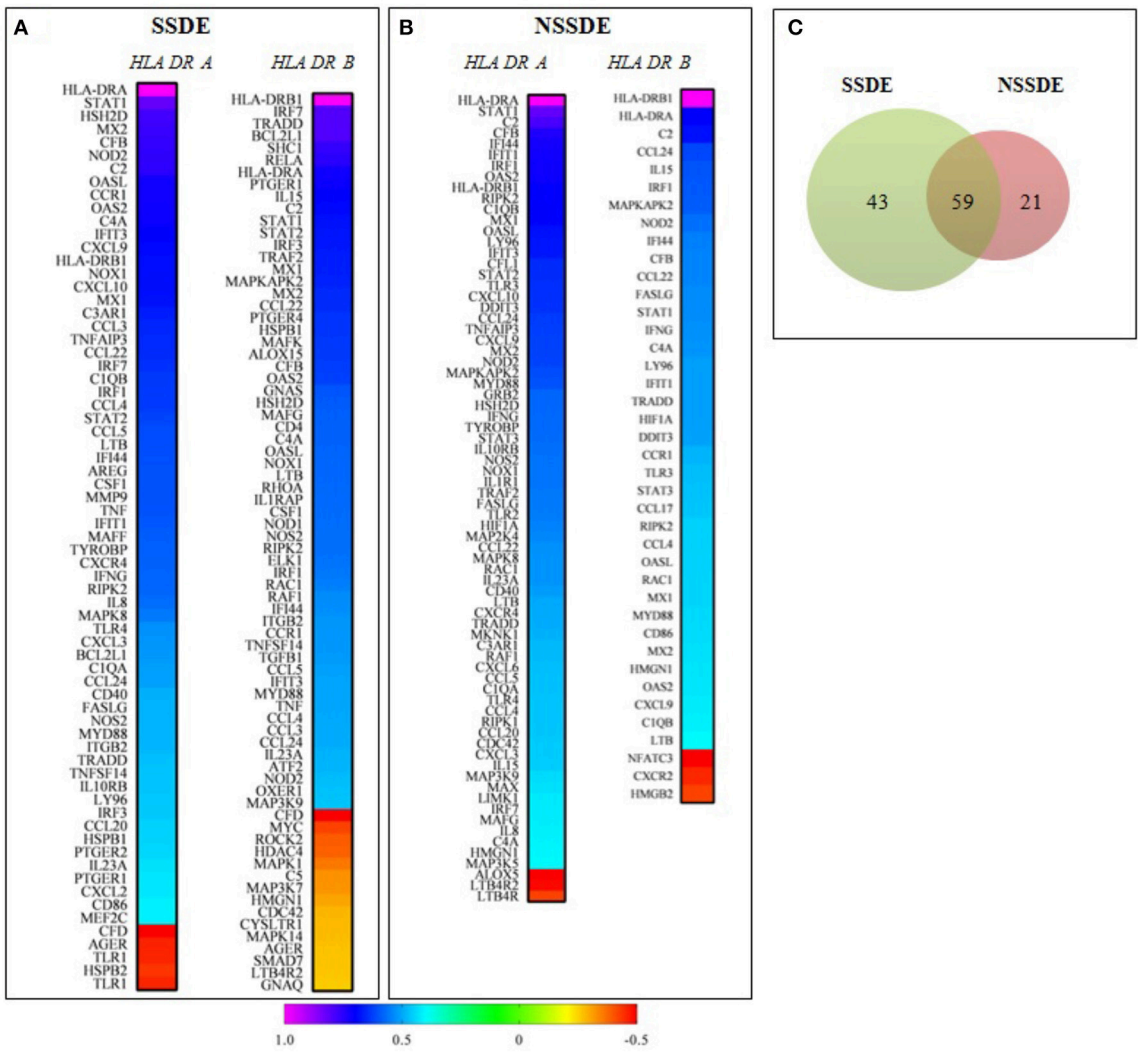
Correlation analysis of genes with *HLA-DRA* and *HLA-DRB1* based on the Spearman's correlation coefficients of genes in SSDE and NSSDE patients. **(A,B)** Represent Heat Map of genes significantly (*P* < 0.05) correlated with both *HLA-DR-A* and *HLA-DRB1*, in a descending manner. **(C)** Venn diagram showing the number of specific and common targets identified in the two groups of patients. SSDE, syndrome Sjögren dry eye; NSSDE, non syndrome Sjögren dry eye.

Following selection of the highly correlated (*R* > 0.8) inflammatory targets with both HLA-DR transcripts, in the whole population (*n* = 88), we wanted to investigate the differential correlated genes between the two major groups of patients; group 1 (SSDE, *n* = 30) and group 2 (NSSDE, *n* = 58) according to their correlation with *HLA-DRA* and *HLA-DRB1*. As upper described, a pairwise Spearman correlation among the 200 genes detected with both HLA-DR receptors *HLA-DRA* and *HLA-DRB1* were applied. Figures 2A,B shows in ascending manner, the genes significantly correlated with both *HLA-DRA* and *HLA-DRB1* in group 1 and group 2. Group 1 (SSDE) present more genes correlated with both *HLA-DR* (*A*/*B1*) than group 2 (NSSDE), with 102 genes vs. 80 genes, respectively. Among the correlated genes, 59 genes were common between both groups while 43 and 21 genes differentially correlated genes with group 1 (SSDE) and group 2 (NSSDE), respectively (Figure 2C).

### Discussion

In this study, we aimed to describe tissue-specific transcriptional networks associated with HLA-DR expression in conjunctival cells of patients with DED stemming from various causes. Presence of HLA-DR is important in inflammatory cells for antigen presentation to CD4 T cells but in epithelial cells, antigen presentation is unlikely and HLA-DR has been considered in the last decades of research in ocular surface diseases mainly as a marker of the inflammatory state, of its level and possibly its mechanisms. The CIs are biological specimens of small size with rare cells compared to blood samples or other tissue samples. The Nanostring technology applied to CIs allows the investigation of numerous targets in only one imprint. CIs provide 3 main cell types from the superficial conjunctival layers with a majority of epithelial cells (more than 90%), followed by goblet cells and inflammatory/immune cells (mainly lymphocytes and dendritic cells). The numbers of these latter cells can also vary considerably according to the level of inflammation, so the source of HLA-DR in relation with a specific function, i.e., activation vs regulation, cannot be fully assessed. Nevertheless, whatever the cell type, these gene expressions reflect the reality of the presence of a local inflammatory stimulation and its relation to HLA-DR. As mentioned above, the pathophysiology of OSD and especially DED is complex and not yet fully understood, but DED is largely recognized as being associated with OS inflammation, resulting in symptoms of eye irritation, alteration of conjunctival and corneal epithelial cells, and corneal barrier dysfunction (1–3, 5, 55).

Considering the complexity of DED diagnosis in terms of clinical criteria, signs and symptoms, many studies, for more than 30 years, have been conducted trying to find reliable biological markers correlated with pathophysiological disease patterns. Here, we have investigated 249 mRNA targets known to be involved in inflammation using the NanoString^®^ technology, considering the extensively reported studies examining the relationships between inflammation and DED (2, 5). Therefore, based on the interest raised by HLA-DR expression in DED patients and the usefulness of measuring this marker in clinical trials for investigating the level of inflammation (13), we focused particularly on the relationships of *HLA-DRA* and *HLA-DRB1* transcripts of HLA-DR heterodimers α and β (23) with inflammatory genes.

**Figure 3:**
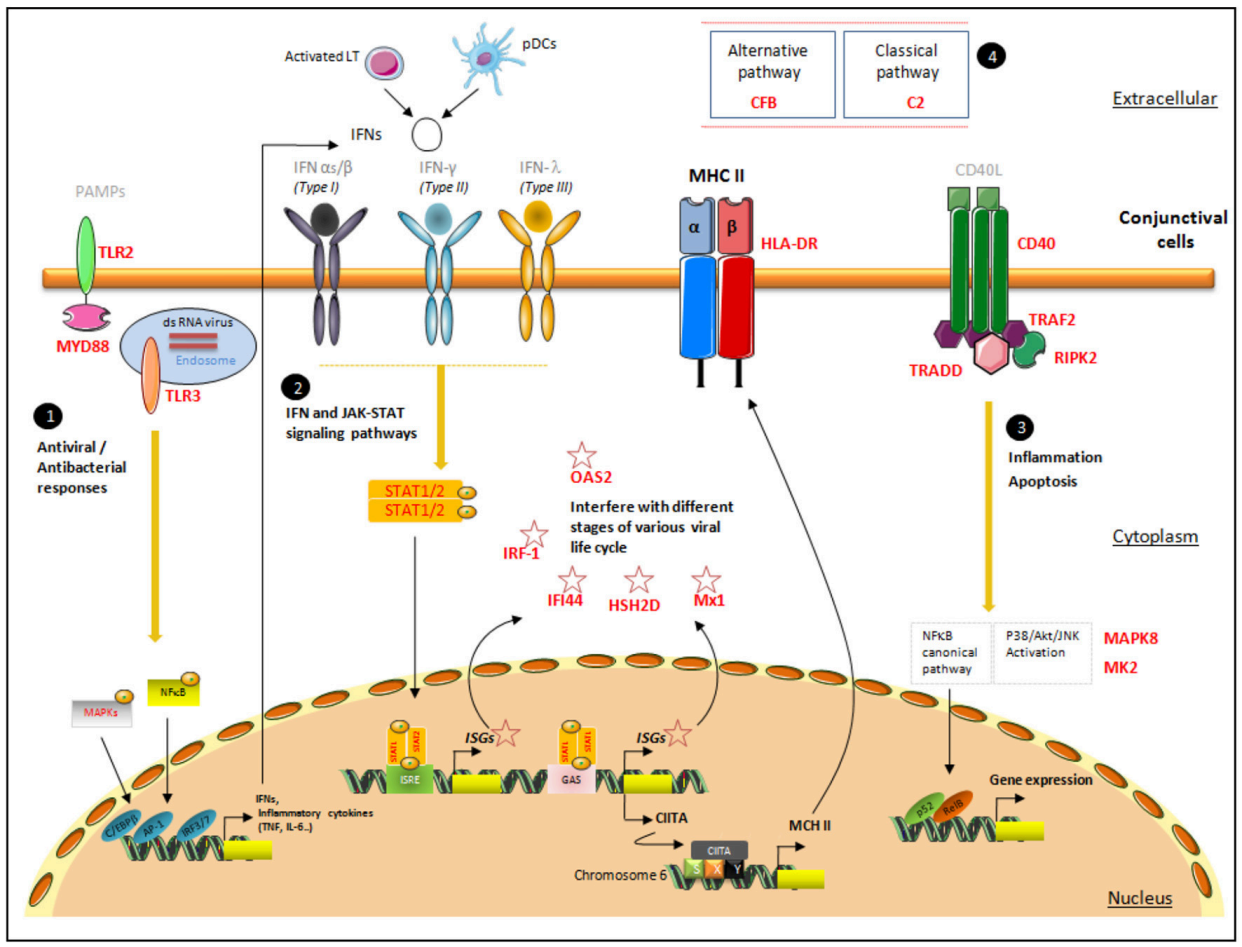
Proposal for a pattern of signaling pathways associated with increased HLA-DR expression in conjunctival cells. This figure presents the localization of the targets identified with their possible interactions in the conjunctiva's spatial microenvironment. The four major signaling responses, in black circles, are mediated by (1) TLR responses, (2) IFN responses, (3) TNF responses, and (4) members of complement pathways. The target genes selected via their high correlation with HLA-DR are mentioned in bold red. The first line of pathogen recognition could be mediated by TLR responses via MAPK and NF-κB to induce inflammatory cytokine responses as INFs. IFNs bind to their receptors and initiate a signaling cascade, involving the JAK-STAT family of transcription factors, which leads to the transcriptional induction of the ISGs (IRF-1, IFI-44, HSH2D, Mx1, and OAS2), class II transactivator (CII-TA), and HLA-DR complex, which will migrate to the membrane. CD40, TRAF2, TRADD, and RIPK2, involved in TNF pathways, promote NF-κB and the MAPK family: MK2 and MAPK8, members of the p38 MAPK and JNK cascades, respectively. Pathogen-associated molecular patterns (PAMPs); CCAAT/enhancer binding protein beta(C/EBPβ); AP-1 transcription factor subunit(AP−1); IFN regulatory factor IRF-3 and IRF-7(IRF3/7); IFN-stimulated response element (ISRE); IFNγ-activated site (GAS); phosphate(P); class II transactivator (CIITA); MHC class II-specific regulatory module (XYS); nuclear factor kappa B subunit 2(p52); NF-κB subunit transcription factor RelB (RelB).

Our results highlighted the expression of a large variety of inflammation-related mediators in conjunctival cells from DED patients. Among the 200 transcripts found to be expressed in conjunctival cells, we focused on those with the strongest correlations with the two HLA-DR transcripts. For that purpose we used very strict criteria, only selecting as markers of interest those with correlation coefficients above 0.8. This allows us to reduce correlation background noise and focus on families of mediators and pathways most likely to play a role in HLA-DR-related cascades of activation. These markers belong to the IFN, TLR, and TNF signaling pathways mediated by STATs and MAPKs, and to the complement and cytokine families (Tables 3A,B). The main interest shown by these results is that in addition to having several targets correlated with HLA-DR, these targets could be integrated into common signaling pathways. Figure 3 summarizes the signaling pathways associated with HLA-DR overexpression in conjunctival cells. Our data confirm the role of IFN, TLR, and TNF pathways, and highlight their close interactions. Indeed, the association of these three families with HLA-DR was previously demonstrated in studies reporting the implication of IFN, TLR, and TNF pathways in molecular processes induced at the cellular level during DED (35, 56, 57).

Nevertheless, the most representative targets were those associated with IFN signaling, represented by IRF-1 (24), IFI-44 (25–27), HSH2D (28, 29), Mx1 (30–32), and OAS2 (33, 34). These ISGs are induced by the three types of IFNs, type I IFNs (IFNα and IFNβ), type II IFN (IFNγ), and type III IFN (IFNλ) (Table 3B). IFNs bind to their cognate receptors and initiate a signaling cascade, involving the JAK family of tyrosine kinases and the STAT family of transcription factors, which leads to the transcriptional induction of the ISGs. Cellular actions of IFNs are largely mediated by the proteins encoded by ISGs, which have important roles in innate immunity against different families of microorganisms. Previous studies reported that the expression of HLA-DR in conjunctival cells might be regulated by IFNγ (57, 58). Indeed, IFNγ induces class II transactivator (CII-TA) expression on different cell types such as epithelial cells, thus stimulating the up-regulation of class II molecules, such as HLA-DR via the SXY module present in all classical MHC class II genes (59).

However, no investigation has yet been conducted on the regulation of these ISGs on HLA-DR expression or on their role in conjunctival cells during DED. Interestingly, IRF-1 was found to be increased in human corneal epithelial cells (HCECs) after a *Pseudomonas aeruginosa* bacterial challenge (60) and seems to be essential for MHC class II gene expression, as described in the mouse macrophage cell line RAW264.7 (61). Moreover, the down-regulation of MHC II gene expression in primary microglial cells by minocycline was reported as mediated by preventing the nuclear translocation of IRF-1 (62). IFI-44 was found up-regulated in the peripheral blood and minor salivary glands of SS patients (63, 64) and displays an anti-proliferative activity in human melanoma cell lines (65). MX1 and OAS2 were also detected in the blood of patients with an autoimmune disease, namely systemic lupus erythematous disease, in which their roles were not totally defined (66). *HSH2D* is able to inhibit IL-2 in Jurkat T cells (67) and its transcripts were found up-regulated in primary airway epithelial cells by IFN type I and III (68). Thus, the association of IRF-1, IFI-44, Mx1, OAS2, and HSH2D with HLA-DR could suggest a possible relationship between viral infection and HLA-DR expression.

The second family group found to be correlated with HLA-DR was TLR members and effectors. This confirms the well-described close relation between IFN and TLR, in the ocular surface inflammatory context, through an autocrine loop that amplifies the IFN response (35, 69). TLRs that recognize pathogen-associated molecular patterns (PAMPs) trigger innate immune responses by activating signaling pathways dependent on the MyD88 adaptors and then induce the expression of type I IFNs, pro-inflammatory cytokines, chemokines, and antimicrobial proteins. Hence, TLR members contribute in the exacerbation of various ocular surface inflammatory processes during infection (36, 37). TLR2 and TLR3 as cell surface and intracellular receptors, respectively (38, 39), are also expressed in human limbal and conjunctival epithelial cells and were demonstrated to play a role in cytokine secretion (69, 70). IFNγ induced TLR2 in *ex vivo* conjunctival cells (36) and TLR3 agonist induced the expression of IFN-β, Mx1 and OAS2 in human corneal epithelial cells (71). The up-regulation of TLR2 and TLR3 may confer an enhanced ability for pathogen recognition, whereas their reduced expression may lead to an inadequate response and therefore an increased risk of infection (35).

In addition to the correlation of TLR and IFN cell signaling members with HLA-DR, the TNF signaling pathway (40), through CD40 (41), TRAF2 (42, 43), TRADD (44), and RIPK2 (45, 46), also seems to be involved in this complex loop of regulation. This latter pathway is mediated by CD40 transduction signal via CD40-TRAF2 to promote nuclear factor-kappa B (NF-κB) and the mitogen-activated protein kinase (MAPK) family (72). TRADD also has a TRAF-binding motif that leads to the recruitment of TRAF1/2, and RIPK2 was described to modulate inflammasome activation through autophagy (45). The interaction between TRADD and RIPK2 with its death domain and C-terminal caspase activation and recruitment domain (CARD), respectively, promotes apoptotic signals (Figure 3).

These results support the idea that the main function of CD40 as a co-stimulatory molecule involved in APC-T-cell interactions is presumably amplified by downstream adaptor proteins, TRAF2, and TRADD. Overexpression of TRAF2 is sufficient to activate NF-κB and AP-1 in the absence of extracellular stimuli (73). Overexpression of TRADD leads to two major TNF-induced responses, apoptosis and activation of NF-κB, by inducing effectors caspase such a caspase-3/7, causing apoptosis (74). We could postulate that these induced signals do not act simultaneously in conjunctival cells, but proceed by sequential steps. Furthermore, previous studies demonstrated that CD40 expression was up-regulated in inflammatory eyes and positively correlated with HLA-DR (75), and was significantly reduced after cyclosporine A, an anti-inflammatory and immunosuppressive treatment (76). This confirms the findings from a previous study showing the association of apoptosis with HLA-DR induction (77), and the key role of apoptosis in the pathogenesis of DED (78). These findings highlight the pivotal role of IFN and TNF responses in the development of a cell-mediated immune response, with a specific interaction of the downstream target with HLA-DR in DED.

As expected, these transduction signals were associated with the well-conserved signaling MAPKs pathways, which promote the expression of inflammatory cytokines and chemokines. In this study, two of them: *MAPKAPK2* (47, 48) and *MAPK8* (49), members of the p38 MAPK and JNK cascades, respectively, were specifically selected according to their correlation with *HLA-DR* transcripts. Interestingly, MAPKAPK2 (MK2) is designed as an emerging therapeutic target, as once inhibited, it is able to block the production of IL-1, TNFα, and other cytokines (79), and MK2-deficient mice showed a reduction of IL-6 and TNFα production (80). More interestingly, a recent study conducted on the effect of the MK2 inhibitor on a mice model of dry eye showed a suppression of cell apoptosis and a decrease of MMP3 and MMP9 in corneal epithelium. Also, SB203580, a selective p38-MAPK inhibitor, showed therapeutic effects on dry eye in a mouse model of Sjögren syndrome (MRL/lpr mice) (81). MAPK8 (JNK1) was also investigated in a mouse model of dry eye, showing an increased level of phosphorylated JNK1/2 in the corneal and conjunctival epithelia (82). Finally, JAK and STAT signaling pathways are closely related to HLA-DR as its expression is modulated in conjunctival cells after treatment of DED patients with tofacitinib (CP-690, 550), a selective inhibitor of the Janus kinase (JAK, JAK1-3) (83).

The remaining targets implicated in inflammatory process and belonging to the STAT family: *STAT1, STAT2, STAT3* (50), chemokines/cytokines: *CCL22* (51) and *IL15* (52), complement and CRP:*C2* (53) and *CFB* (54) were briefly described in Table 3B.

The second part of this study gives a brief overview of correlated genes specifically associated with the two major pathological groups as SSDE and NSSDE. As expected the SSDE group presents more genes correlated with *HLA-DR* than the NSSDE group. More interestingly, 43 and 21 selective genes are only associated with SSDE and NSSDE respectively. These results highlight that the conjunctival profile of HLA-DR correlated genes with SSDE and NSSDE patients present some differences in molecular inflammatory responses.

Among these selective genes for SSDE, *MMP9* (*R* = 0.6), Transforming growth factor beta *(TGFB)* (*R* = 0.46), and *CCL3* (*R* = 0.44), present a particular interest to distinguish inflammatory responses and for therapy management especially in SSDE group. TGF-β is known to regulate the immune system, and enhance the synthesis and deposition of extracellular matrix, during wound repair (84). As previously described, level of TGF-β1 mRNA within the conjunctival epithelium of patients with SS is higher when compared to non-DE controls (9) and its bioactivity increases in tears (85). MMP-9 has important roles in the DED inflammatory process (86), likewise tears and saliva of SS patients contain high levels of MMP-9 (9). Finally, tear expression of CCL3 was reported to be increased in DE patients compared to healthy control subjects, especially in those with SS (87) and CCR5 receptor of CCL3 is positively correlated with HLA-DR in conjunctival cells of patients (88); it is also known also that CCL3 shows a significant distribution in salivary of pSS compared to non-SS sicca (89). More interestingly among the genes correlated with HLA-DR in both SS and NSS groups, two of them present an inverse correlation with *HLA-DR* in the two groups. As: High-mobility group nucleosome-binding protein 1 *(HMGN1)* (*R* = −0.45 in SSDE) and (*R* = 0.43 in NSSDE) followed by Cell division cycle 42 (*CDC42)* (*R* = −0.43 in SSDE) and (*R* = 0.44 in NSSDE). HMGN1 is a member of the HMGN family of proteins that bind specifically to nucleosomes and is known to affect chromatin structure and function, including transcription and DNA repair (90). It is also described to act as a novel alarmin critical for LPS induced development of innate and adaptive immune response (91). Cdc42 is a small GTPase of the Rho family, has pivotal functions in cell migration and proliferation, and is known to be essential for human T-cell development, where loss of expression induces apoptosis and reduced proliferation (92). Even if these genes display a mild correlation of *R* = 0.4, the mirror suggested effect, could be interesting for further investigation concerning the molecular responses associated with HLA-DR in an autoimmune and non-autoimmune context of DED. Indeed, at this stage of the investigation, we cannot hypothesize to a functional role corresponding of this loss of expression in presence of high level of HLA-DR in SSDE, but we only point out the deleterious effect of this target in presence of high level of inflammation especially in case of severe DED. This could be also helpful in managing SSDE and NSSDE with anti-inflammatory therapy (93).

In summary, this original work highlights the implication of a large set of inflammatory mediators in DED with the same tendency as with two HLA-DR forms (A and B1). All these identified target genes could work in concert in a spatial microenvironment to efficiently promote cell recruitment and maintain an inflammatory state in conjunctival cells (Figure 3). This combination of genes associated with HLA-DR corresponds to biologically meaningful modules in a network, which could become future candidates for drug development. These outcomes also support the assumption that inflammation is a core pathophysiological process in DED, maintaining a vicious circle of inflammation, and a self-perpetuating cycle ensues (4). Moreover, the nCounter analysis system from NanoString^®^ technologies applied on CIs is a reliable tool for multiplexed gene expression analysis of the inflammatory biomarkers in DED, and more generally other OSDs, especially when only tiny samples are available. This tool was applied in ophthalmology for the first time and is a powerful tool for the detection of specific molecular targets. This methodology expands the repertoire of approaches for expression profiling and offers several advantages over existing technologies, as it requires less sample material, has no enzymatic bias, and provides a direct digital readout. As yet, however, no single protein or panel of markers has been shown to discriminate between the major forms of DED. The gene expression profiling could contribute to understanding more fully the discrepancy between signs and symptoms in DED (94) and the failure of some therapies. Although this transcriptomic platform is still in its early stages in clinical use, especially in the cancer biology field (95, 96), it is expected that NanoString^®^-based inflammatory expression panels can play a more important role in the future for classifying DED patients and predicting their response to different treatment strategies. Finally, these molecular actors, selected upon a high level of correlation with HLA-DR, could improve our knowledge on the pathophysiology of DED, for a better understanding of the underlying regulation loop and to define their role in conjunctival cells and the ocular surface.

## Author contributions

CB, FB-B, KK, PD, and J-SG designed the study and planned the experiments. CB, HL, and GR supervised the clinical part for the recruitment of the patients and clinical data analysis. KK and MD performed experiments and transcriptomic data interpretation. FB-B, KK, and PD wrote the manuscript. CB, FB-B and SMP aided in interpreting the results and worked on the manuscript. All authors discussed the results and commented on the manuscript.

### Conflict of interest statement

J-SG and PD are Santen SAS employees. CB is consultant for Santen SAS. The remaining authors declare that the research was conducted in the absence of any commercial or financial relationships that could be construed as a potential conflict of interest.

## Footnotes

^1^https://www.nanostring.com/products/gene-expression-panels/ncounter-inflammation-panels
